# Correlation between bicuspid aortic valve fusion phenotype and aortic arch morphology using MRI

**DOI:** 10.1186/1532-429X-14-S1-P91

**Published:** 2012-02-01

**Authors:** Alexander Turin, Bryce Merritt, Jennifer A Febbo, Michael Markl, James Carr

**Affiliations:** 1 Northwestern University, Chicago, IL, USA

## Summary

The purpose of this study is to use magnetic resonance angiography (MRA) to evaluate the relationship between different patterns of bicuspid aortic valve (BAV) fusion and aortic arch morphologies, as characterized by the carotid-subclavian artery (CSA) index - of the aortic diameter at the left subclavian artery to the distance between the left carotid artery and the left subclavian artery - and aortic arch height and width dimensions.

## Background

BAV is associated with aortic aneurysms, various aortic root morphologies, and morphological changes in the transverse arch [1]. Although height and width parameters have been used to classify different shapes of the thoracic aorta, the effects of BAV on these dimensions have not been characterized [2]. Moreover, there has been little investigation of the individual BAV fusion phenotypes with the associated complications of BAV [3]. A greater understanding of the association between BAV fusion types and disease will help to shed light on the nature of the hemodynamic effects of the inherent mechanism involved.

## Methods

A total of 191 patients with previously diagnosed BAV and 26 age- and sex-matched controls with tricuspid aortic valves (TAV) who had undergone MRA of the chest were retrospectively identified. Measurements of the carotid-subclavian artery index as well as the height and width of the aortic arch were taken and compared to BAV fusion phenotypes.

## Results

The CSA index values did not differ significantly across BAV fusion patterns, although there was significant variation in the mean diameter of the aortic arch at the left subclavian artery among all groups and an increased carotid-subclavian artery distance in patients with left-non (L-N) coronary cusp fusion. All BAV fusion types are also associated with an increase in aortic arch height. Lastly, patients with L-N fusion were also shown to have a significantly increased aortic arch height-width ratio (0.79 vs. 0.58, p = 0.002) compared to control.

## Conclusions

Bicuspid aortic valve fusion phenotypes have an influence on aortic arch morphology, and the differences in the aortic arch measurements both within and among groups suggest that a hemodynamic factor is present in aortas with BAV.

## Funding

None.
